# The association of interieukin-6 polymorphism (rs1800795) with microvascular complications in Type 2 diabetes mellitus

**DOI:** 10.1042/BSR20201105

**Published:** 2020-10-16

**Authors:** Jieyuan Cui, Xiaolin Zhang, Cheng Guo, Lin Zhang

**Affiliations:** 1 Department of Nephrology and Immunology, Children’s Hospital of Hebei Province, Shijiazhuang 050031, China; 2 Department of Pediatric, The Third Hospital of Hebei Medical University, Shijiazhuang 050051, China; 3 Department of Epidemiology and statistics, School of Public Health, Hebei Medical University, Hebei Province Key Laboratory of Environment and Human Health, Shijiazhuang 050020, China

**Keywords:** Diabetic microvascular complications, Gene polymorphism, Interleukin-6 gene, rs1800795, Type 2 diabetes mellitus

## Abstract

Objectives: To evaluate the effects of the single-nucleotide polymorphism (SNP) rs1800795 in interieukin-6 (IL-6) gene on diabetic microvascular complications of Type 2 diabetes mellitus (T2DM), using statistical meta-analysis.

Methods: Literature pertaining to the relationship between the SNP rs1800795 and microvascular complications of T2DM including diabetic retinopathy, diabetic nephropathy, diabetic neuropathy and foot disease was retrieved from PubMed, Web of Science Knowledge and SinoMed databases. Original information was analyzed using Stata 12.0, including meta-analysis statistics, test for heterogeneity, evaluation of publication bias and sensitivity. Subgroup analysis was conducted to assess the effect of specific factors on the corresponding results.

Results: In total, 14 eligible articles were obtained. The SNP rs1800795 in IL-6 gene is not correlated with risk of microvascular complications in T2DM. Among the original literature, a genetic model (OR = 1.071, 95% CI: 0.681–1.685, *P*=0.767), an allelic genetic model (OR = 1.010, 95% CI: 0.959–1.063, *P*=0.703), a heterozygote genetic model (OR = 1.107, 95% CI: 0.916–1.339, *P*=0.292), a dominant genetic model (OR = 1.108, 95% CI: 0.885–1.387, *P*=0.372), and a recessive genetic model (OR = 0.978, 95% CI: 0.646–1.478, *P*=0.917) were included respectively. In the subgroup analysis by types of diabetic microvascular complications, we found no correlation between the SNP rs1000795 polymorphism and complications of T2DM in either the homozygote genetic model or the allelic genetic model (*P*<0.05).

Conclusion: Our results demonstrate that rs1800795 polymorphism in IL-6 gene is not correlated with the susceptibility of microvascular complications of T2DM.

## Introduction

Diabetes mellitus (DM) is a chronic metabolic disease. Over 382 million people worldwide have DM, the prevalence of Type 2 diabetes mellitus (T2DM) in children and adolescents is on the rising around the world, in parallel with the increase in the rate of obesity [1]. DM might become the seventh leading cause of death in humans by 2030 [2]. DM is associated with microvascular and macrovascular complications, which are considered one of the major causes of morbidity and mortality. Chronic complications, especially microvascular complications (diabetic retinopathy, nephropathy, foot disease, and neuropathy) are the major dangerous outcome of this disease. Chronic inflammatory processes are involved in the development of diabetic microvascular complications. Inflammatory cytokines including interleukin-6 (IL-6) play an important role in the pathogenesis of T2DM and its complications [3].

The single-nucleotide polymorphism (SNP) of IL-6 gene has attracted more and more attention recently. Significant sequence variation of IL-6 174G/C gene is widely distributed at varying serum IL-6 levels in genetically susceptible individuals [4]. G/C polymorphism at the position-174 in IL-6 promotor region (rs1800795) has been found to correlate with retinopathy, nephropathy [5], increased albumin-to-creatinine ratio as well as poor glycemic control and hyperlipidemia in Type 1 diabetes mellitus (T1DM) [6]. However, the results of current studies on the involvement of this SNP rs1800795 in diabetic complications in T2DM are controversial. Whether IL-6 gene polymorphisms rs1800795 may serve as genetic predictors for the progression of complications in T2DM, and contribute to the identification of patients with high risk of diabetic complications, thus helping them with tailored treatments remains unclear. We conducted first a meta-analysis of eligible case–control studies of SNP rs1800795 and the risk of diabetic microvascular complications in T2DM to assess the questions raised above.

## Materials and methods

### Retrieval strategy

We intended to summarize data on the correlation between SNP rs1800795 (174G/C) in IL-6 gene and the risk of microvascular complications including diabetic retinopathy, nephropathy, neuropathy, or foot disease in T2DM. In this field, we conducted a literature search in PubMed, Web of Science Knowledge and SinoMed databases for related studies from January 1, 2000 to December 1, 2019. The search terms used are: (1) ‘SNP’ OR ‘single gene polymorphism’ OR ‘mutation’; (2) ‘rs1800795’ OR ‘Interieukin-6’ OR ‘IL-6 174G/C’; (3) ‘type 2 diabetes’ OR ‘type 2 diabetes mellitus’ OR ‘T2DM’; (4) ‘diabetic retinopathy’ OR ‘diabetic nephropathy’ OR ‘diabetic neuropathy’ OR ‘diabetic foot disease’ OR ‘diabetic complications’ OR ‘diabetic microvascular complications’. We made an effort to contact authors if some relevant data were needed. We have also done a bibliographic search for any other relevant studies.

This meta-analysis was undertaken in accordance with PRISMA guidelines (http://prisma-statement.org/PRISMAStatement/Checklist).

### Inclusion criteria

For inclusion, all data were used as measurement data in our study, so data from the original literature needed to be clearly described. Studies of high-quality design incorporating highly accurate research methods were selected for analysis. Inclusion criteria are: (1) single-nucleotide polymorphism rs1800795 in IL-6 gene 174G/C position was assessed; (2) the final outcome was T2DM with or without microvascular complications; (3) the number of different genotypes and risk of complications respectively were provided; (4) clear presentation of units for data; (5) studies were obtained by means of standard formula.

Two investigators extracted the data independently, discrepancies were resolved by discussion.

### Study subjects

Overall, 14 independent original articles (from 2000 onwards) including 4934 subjects and 9868 genetic locus were retrieved, based on relevant data on the effects of IL-6 gene promoter polymorphism (G/C) at position-174 variations (SNP rs1800795) on frequencies in complications of T2DM.

## Meta-analysis statistics

Meta-analysis and statistical analyses were conducted using the statistical software Stata 12.2. *P* values <0.05 were considered statistically significant. The chi-square test was utilized to assess the offset of frequencies of IL-6 polymorphisms from the expected values under the Hardy–Weinberg equilibrium (HWE) among T2DM patients with or without microvascular complications. Odds ratio (OR) was used as measure correlation between SNP rs1800795 in IL-6 gene 174G/C and risk of diabetic complications. The association between rs1800795 and the risk of diabetic complications was measured by the pooled OR with its corresponding 95% CI. We assessed the association between rs1800795 polymorphism and the susceptibility to complications of T2DM. The genetic models employed were exhibited as follows: the allelic genetic model (G vs. C), the homozygote genetic model (GG vs. CC), the heterozygote genetic model (GG vs. GC), the dominant genetic model (CC+CG vs. GG), and the recessive genetic model (CC vs. GG+GC) [7].

The statistical heterogeneity of the studies was calculated using the chi-square-based *Q* test and *I*-squared index. If *I*-squared index was >50% and *P* value was <0.1, the study would be considered as significant heterogeneity, and in consequence the random-effected model would be used; On the contrary, if *I*-squared index was proved to be <50% with the *P* value of >0.1, the study would be considered as not of significant heterogeneity, and the fixed-effects model would be selected. To assess the impact of possible factors on pooled effect size and heterogeneity, subgroup analyses were performed.

Publication bias was assessed using the funnel plot method and Begg’s test, the level of *P*<0.05 was considered as statistically significant. Sensitivity analysis was conducted to test the influence of methodological quality by removing any low-quality studies from the meta-analysis. It was performed to determine the effect of each individual research on the pooled outcomes. As a measurement tool for investigation the quality of studies, ‘leave one out’ was used to evaluate the influence of the respective data on the integrated data by excluding one single data set from the pooled analyses one at a time.

Study quality was assessed using Newcastle–Ottawa Scale (NOS) ranging from a low of 0 to a high of 10 with the higher score representing higher quality. The cut-off values of NOS < or =5 suggested low-quality study while NOS >6 suggested high-quality study.

## Result

Our study was intended to evaluate the relationship between the single-nucleotide polymorphism rs1800795 in IL-6 gene 174G/C and the susceptibility of diabetic microvascular complications in T2DM.

### Characteristics of eligible studies

**Figure 1:**
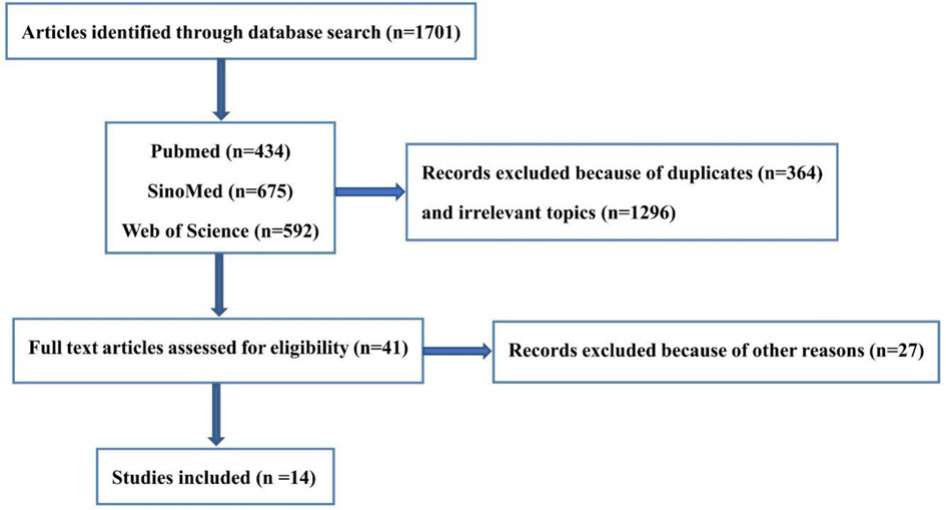
Study selection flowchart

We identified 20 databases from 14 studies with diabetic microvascular complications of T2DM including diabetic retinopathy, diabetic nephropathy, diabetic foot disease, and diabetic neuropathy that met the inclusion criteria of this meta-analysis [8]. Figure 1 shows the flowchart of the study selection, by means of which 1701 articles were identified in the databases including 434 articles in Pubmed, 675 in SinoMed and 592 in Web of Science, respectively. About 41 articles were assessed at full-text level and 14 articles were included finally in this meta-analysis. These 14 articles included 2 low-quality studies and 12 high-quality studies (Table 1). The characteristics of articles included in this meta-analyses are listed in Table 1. The primary studies included in this meta-analyses were published between 2009 and 2019. There was no any effort to contact authors for relevant data, and the bibliographic search found no other relevant studies.

**Table 1:** Characteristics of including studies

Study	Author	Ethnicity	Year	Case/control	M/F	NOS	Complications
1	Rudofsky [19]	Germany	2009	216/282	/	5	Diabetic retinopathy, nephropathy, and foot disease
2	Paine [9]	India	2012	253/340	261/232	6	Diabetic retinopathy
3	Dhamodharan [10]	India	2015	270/139	261/148	7	Diabetic retinopathy, neuropathy, and foot disease
4	Lu [24]	Han	2017	215/207	/	7	Diabetic retinopathy
5	Erdogan [21]	Turkey	2017	50/35	42/43	6	Diabetic retinopathy, neuropathy, and foot disease
6	Abrahamian [20]	Austria	2007	66/75	/	7	Diabetic nephropathy
7	Ng [25]	America	2008	294/168	/	9	Diabetic nephropathy
8	Karadeniz [22]	Turkey	2014	43/43	/	6	Diabetic nephropathy
9	Zambrano-Galvan [29]	Mexico	2015	70/60	/	6	Diabetic nephropathy
10	Chang [16]	Tai wan	2016	143/424	269/298	9	Diabetic nephropathy
11	Neelofar [26]	Indian	2017	50/50	/	8	Diabetic nephropathy
12	Papaoikonomou [30]	Greece	2014	153/258	208/203	5	Diabetic nephropathy
13	Hameed [31]	India	2018	448/414	/	6	Diabetic nephropathy
14	Fathy [17]	Kuwaiti	2019	61/107	/	6	Diabetic nephropathy

### Findings from meta-analysis

**Figure 2:**
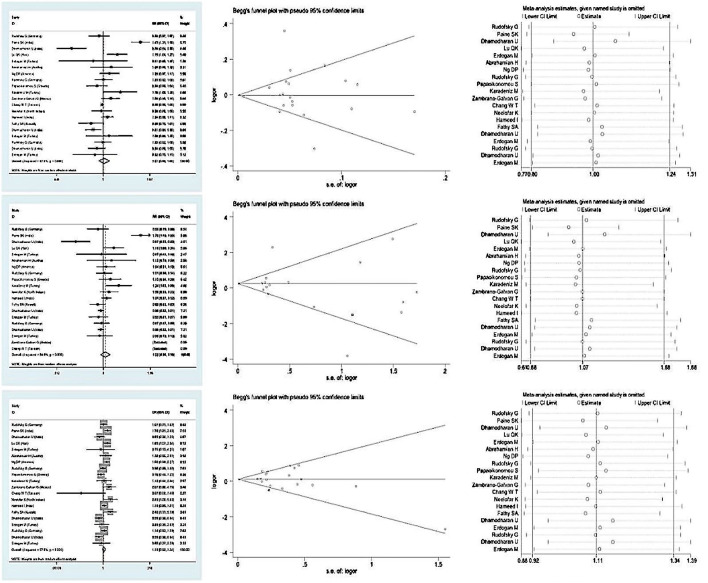

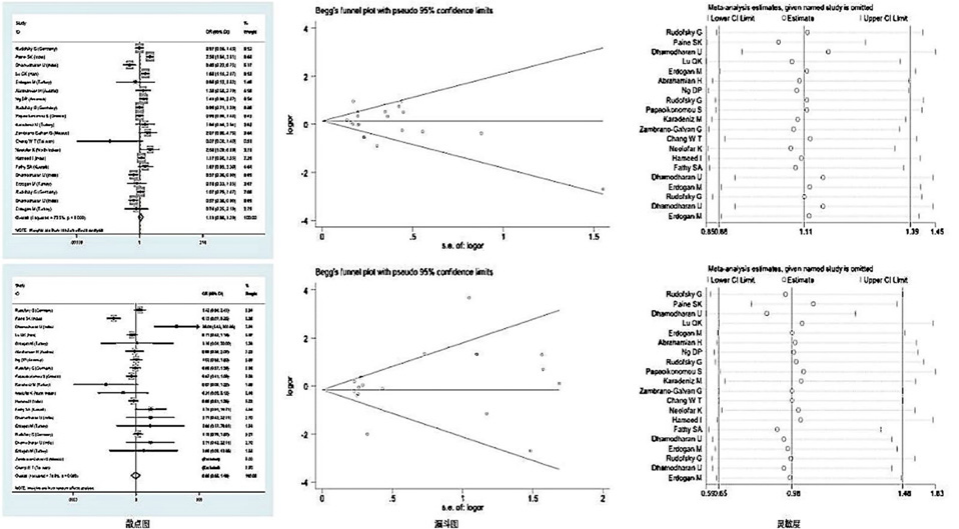
Forest plot for meta-analysis of association between IL-6 -174G/C polymorphism with risk of complications in Type 2 diabetes in subgroup analysis Begg’s funnel plot showing the publication bias and results of sensitivity analysis showing by measurement tool of leave one out in subgroup analysis.

The association between rs1800795 polymorphism in IL-6 gene and the susceptibility to diabetic microvascular complications of T2DM under five genetic models was assessed. We found that there was a lack of association between the risk of diabetic microvascular complications in T2DM and a homozygote genetic model (OR = 1.071, 95% CI: 0.681–1.685, *P*=0.767), an allelic genetic model (OR = 1.010, 95% CI: 0.959–1.063, *P*=0.703), a heterozygote genetic model (OR = 1.107, 95% CI: 0.916–1.339, *P*=0.292), a dominant genetic model (OR = 1.108, 95% CI: 0.885–1.387, *P*=0.372), and a recessive genetic model (OR = 0.978, 95% CI: 0.646–1.478, *P*=0.917), respectively (Table 2 and Figure 2).

**Table 2:** Overall analysis between genotypes and allele frequency with complications of T2DM

Genetic model		Case (*n*)	Control (*n*)	*P* (value)	OR (95% CI)	*I*^2^ (%)	*P* for heterogeneity
G vs. C	G	4147	5599	0.703	1.010	87.0	<0.010
	C	1601	2315		(0.959–1.063)		
GG vs. CC	GG	1548	2096	0.767	1.071	77.0	<0.010
	CC	278	477		(0.681–1.685)		
GG vs. GC	GG	1548	2096	0.292	1.107	57.6	<0.010
	GC	1045	1427		(0.916–1.339)		
CC+CG vs. GG	CC+CG	1323	1904	0.372	1.108	73.5	<0.010
	GG	1548	2096		(0.885–1.387)		
CC vs. GG+GC	CC	278	477	0.917	0.978	74.8	<0.010
	GG+GC	2593	3523		(0.646–1.478)		

In the subgroup analysis by types of diabetic microvascular complications, we also found that rs1000795 polymorphism was independent of the susceptibility of the four complications mentioned above in T2DM (*P*>0.05), both the homozygote genetic model and the allelic genetic model, with a homozygote genetic model (OR = 0.952, 95% CI: 0.232–3.911, *P*=0.945) and an allelic genetic model (OR = 1.009, 95% CI: 0.499–2.039, *P*=0.981) in diabetic retinopathy, a homozygote genetic model (OR = 1.229, 95% CI: 0.939 1.607, *P*=0.133) and an allelic genetic model (OR = 1.062, 95% CI: 0.881–1.282, *P*=0.527) in diabetic nephropathy, a homozygote genetic model (OR = 0.230, 95% CI: 0.039–1.353, *P*=0.104) and an allelic genetic model (OR = 0.808, 95% CI: 0.434–1.501, *P*=0.499) in diabetic foot disease, and a homozygote genetic model (OR = 0.825, 95% CI: 0.519–1.311, *P*=0.415) and an allelic genetic model (OR = 0.919, 95% CI: 0.758–1.115, *P*=0.996) in diabetic neuropathy, respectively (Table 3 and Figure 2).

**Table 3:** Subgroup analysis of genotypes and allele frequency with complications of T2DM

Complication	Genetic model		Case (*n*)	Control (*n*)	*P* (value)	OR (95% CI)	*I*^2^ (%)	*P* for heterogeneity
Diabetic retinopathy	GG vs. CC	GG	352	504	0.945	0.952	92.5	<0.010
		CC	83	196		(0.232–3.911)		
	G vs. C	G	959	1499	0.981	1.009	94.7	<0.010
		C	421	883		(0.499–2.039)		
Diabetic nephropathy	GG vs. CC	GG	673	1070	0.133	1.229	0.0	0.944
		CC	337	492		(0.939–1.607)		
	G vs*.* C	G	1683	2672	0.527	1.062	48.2	0.043
		C	525	794		(0.881–1.282)		
Diabetic foot disease	GG vs. CC	GG	125	101	0.104	0.230	0.0	0.944
		CC	7	1		(0.039–1.353)		
	G vs. C	G	359	164	0.499	0.808	57.5	0.125
		C	123	64		(0.434–1.501)		
Diabetic neuropathy	GG vs. CC	GG	230	315	0.415	0.825	0.0	0.427
		CC	41	62		(0.519–1.311)		
	G vs. C	G	666	868	0.996	0.919	0.0	0.458
		C	288	402		(0.758–1.115)		

### Heterogeneity assessment

A significant between-study heterogeneity was found (*I*^2^ = 57.6–87.0%, *P*<0.01) (Table 2). We used subgroup analysis to find out the sources of heterogeneity through random-effects. The subgroup analysis by types of diabetic complications resulted to resolve the heterogeneity and the test of between-subgroup heterogeneity was significant (*I*^2^ = 77.0–83.2%, *P*<0.01; Table 3).

### Publication bias and sensitivity analysis

Begg’s test was carried out to assess the potential publication bias in the present study and the result showed that significant publication bias was not observed (*P*>0.1). There were no asymmetries in Funnel’s plots in the current analyses (Figure 2).

Sensitivity analysis was implemented to evaluate the results and overall meta-analysis and subgroup analysis was performed for sensitivity analysis according to measurement tool. We found that all of the results remained relatively stable by excluding individual studies.

## Discussion

As the prevalence of T2DM has risen to epidemic scale worldwide, diabetic complications have now become one of the most challenging health problems. The diabetic microvascular complications such as diabetic retinopathy, nephropathy, neuropathy, or foot disease have always been the major dangerous outcomes of the disease, which contribute to disabilities and the high mortality rate in T2DM patients [1]. There is no diagnostic tool that can predict the high risk of developing diabetic microvascular complications before any damage is presented in a patient. In recent years, tests of genetic susceptibility to T2DM and its complications have been conducted. Genetic biomarkers as earlier predictable biomarkers may provide a new insight into the prediction and diagnosis of diabetic complications [9,10].

Chronic low-grade activation of the immune system has been implicated in the pathogenesis of T2DM. Interleukin-6 (IL-6) has been established to play the role of inflammatory cytokine in the pathogenesis of T2DM. Dysregulation of IL-6 signaling and IL-6 itself has been implicated in the etiology of several autoimmune and inflammatory diseases including T2DM [11]. The elevated circulating level of IL-6 is an independent predictor of T2DM, patients with T2DM have significantly higher level of IL-6 compared than those without diabetes [12]. Studies have confirmed that IL-6 and its expression are associated with insulin resistance and β-cell dysfunction, diabetic retinopathy and obstructive sleep apnea hypopnea syndrome (OSAHS) in T2DM [13–15]. However, IL-6 level in T2DM patients with diabetic microvascular complications is considered to be no higher than in patients without complications [16,17].

SNP rs1800795 (-174G/C) of IL-6 gene in T2DM was first reported in 2003 showing that GG phenotype was a genetic determination of inflammation in the development of T2DM [18]. Significant sequence variation of IL-6 174G/C gene was widely distributed at varying frequencies in T2DM and healthy persons; this risk allele was also responsible for increased IL-6 serum level in genetically susceptible individuals [4]. However, the involvement of this SNP rs1800795 in diabetic complications in T2DM is unclear or controversial [5,19]. In order to further conform the roles of IL-6 rs1800795 in the predication the risk of diabetic microvascular complications in T2DM, we conducted here a meta-analysis of eligible case–control studies, the association between rs1800795 polymorphism in IL-6 gene and risk susceptibility to diabetic microvascular complications of T2DM under five genetic models was assessed.

Our results showed that there was no correlation between risk of diabetic microvascular complications in T2DM and a homozygote genetic model (OR = 1.071, 95%CI: 0.681–1.685, *P*=0.767), an allelic genetic model (OR = 1.010, 95% CI: 0.959–1.063, *P*=0.703), a heterozygote model (OR = 1.107, 95% CI: 0.916–1.339, *P*=0.292), a dominant genetic model (OR = 1.108, 95% CI: 0.885–1.387, *P*=0.372), and a recessive model (OR = 0.978, 95% CI: 0.646–1.478, *P*=0.917), respectively. In the subgroup analysis by types of diabetic microvascular complications, we also found that rs1000795 polymorphism was independent of the susceptibility of the four complications mentioned above in T2DM patients (*P*>0.05), in both the homozygote genetic model and allelic genetic model. Our results were consistent with most other studies. The study of Rudofsky et al. [19] did not find any association between IL-6 -174G/C polymorphism and diabetic neuropathy or diabetic nephropathy in T2DM, which was supported by some other studies [16,17,20]. The study of Paine et al. [9] indicated that the promoter polymorphism of IL-6 was not a potent risk factor for the pathogenesis of proliferative diabetic retinopathy. Other studies [10,21] showed that there was no association between IL-6 -174G/C and diabetic foot disease risk in T2DM. However, there are contrary studies. Karadeniz et al. [22] evaluated the -174 G>C polymorphism of the IL-6 gene in T2DM with diabetic nephropathy in 43 cases of T2DM and 43 cases of T2DM with diabetic nephropathy and 340 healthy normal controls, the result of which showed that the frequency of the polymorphic G allele was 83.9% in diabetic patients with nephropathy versus 70.9% in those without nephropathy (*P*=0.039), the author suggested that the -174 G>C polymorphism of the IL-6 gene might be an independent risk factor for diabetic nephropathy in Turkish T2DM. The limitation of the present study was that the sample was too small. The study of Chen et al. [7] indicated that IL-6 rs1800795, rs1800796 and rs1800797 might play important roles in diabetic nephropathy development while IL-6 rs2069837 and rs2069840 might not be related to diabetic nephropathy, but it is a pity that the data analyzed in the present study included both T1DM and T2DM, and the results of T2DM were not analyzed separately. Libra et al. [23] also claimed that the GG genotype of IL-6 C-174G polymorphism might promote peripheral arterial disease development among individuals with T2DM by inducing increased release of IL-6 but without normal controls. Lu et al. [24] concluded that IL-6 genotypes of rs1800795 GC and rs1800796 GG might point to a relatively high risk for T2DM patients suffering from proliferative diabetic retinopathy in a Chinese population and which was associated with elevation of IL-6 expression in both mRNA and protein, but the sample was also too small (215 cases of T2DM with proliferative diabetic retinopathy and 207 cases of T2DM with a normal retinal function) and without normal controls. It was interesting that two studies showed that the -174G>C polymorphism may be associated with the risk of chronic renal failure [25,26]. It is a pity that we could not get data on renal function or analyze this phenomenon in our meta-analysis.

Numerous studies have provided clear evidence that IL-6 C-174G single-nucleotide polymorphism was a risk factor in Type 2 diabetes and contributed to higher serum interleukin-6 level among the participants [4,27,28]. We suggested that the SNP rs1800795 might be related to developing T2DM, but it might be of no major functional relevance in influencing the development and progression of diabetic microvascular complications. Furthermore, this could be due to different haplotypes with respect to other possible variants in the IL-6 promoter, which interfere with the promoter activity. Alternatively, sample size studied was insufficient to analyze associations of IL-6 polymorphism with diabetic microvascular complications. In addition, the target population we selected was T2DM patients. There may be significant differences in this SNP between T2DM patients and non-diabetic patients, but there is no significant difference among T2DM patients with or without complications in individuals. Finally, besides the IL-6 rs1800795, other SNPs of IL-6 such as rs1800796, rs1800797, rs1524107, rs2069837, rs2069840, etc. might also play important roles in the development of diabetic nephropathy or T2DM [7,16,29]. For example, Chang [16] suggested that IL-6 gene polymorphisms rs1800796 and rs1524107 might serve as predictors of progression of nephropathy in Chinese T2DM. This also needs further studies especially meta-analysis to prove.

## Conclusion

In summary, comprehensive meta-analysis was performed and revealed no correlation between the SNP rs1000795 in IL-6 gene and diabetic microvascular complications in patients with T2DM. The results obtained in our study suggested that screening SNP rs1800795 may be helpless in identifying the high risk of developing diabetic microvascular complications in patients with T2DM. However, our findings still need to be confirmed by further large sample, multicenter and high-quality studies to provide theoretical basis. Our conclusion requires updated meta-analysis with a large sample size.

## Limitations

Our review has some limitations that should be mentioned. First, the methodological quality of the published literature should be noted, as only 12 of the 14 studies were categorized as high-quality ones (Table 1). Second, limited reference available for meta-analysis may lead to bias in the results. Third, the target population we selected was T2DM patients, there may be significant differences in this SNP between T2DM patients and non-diabetic patients. Finally, some factors such as the small number of participants in some studies, could explain the heterogeneity in our results. These limitations reduced the reliability and strength of the conclusions. It is possible that these studies of our meta-analysis containing systematic biases may have confounded results.

## Abbreviations

DM: diabetes mellitus
IL-6: interleukin-6
NOS: Newcastle–Ottawa Scale
SNP: single-nucleotide polymorphism
T1DM: Type 1 diabetes mellitus
T2DM: Type 2 diabetes mellitus
